# Engagement and Immersion in Digital Play: Supporting Young Children’s Digital Wellbeing

**DOI:** 10.3390/ijerph181910179

**Published:** 2021-09-28

**Authors:** Kelly Bittner

**Affiliations:** School of Education, Macquarie University, Sydney 2109, Australia; kelly.johnston@mq.edu.au

**Keywords:** digital technology, young children, self-regulation, wellbeing, flow theory

## Abstract

For many families, young children’s engagement with screen-based technology is an ongoing concern in terms of physical, social and cognitive development. They are uneasy with the difficulty children have disengaging from screens and concerned that this behavior is obsessive or a sign of addiction. However, technology is recognized as having a “rightful role” in early childhood contexts. This scoping paper reports on a review of literature relating to digital play for children aged birth to five years, with the aim of further understanding digital wellbeing. Csikszentmihalyi’s flow theory serves as a theoretical framework for understanding why many young children enjoy digital play and become deeply engaged, with a disconnect between how young children and adults perceive digital play. Concerns about children’s deep immersion with digital play are interrogated to understand the connections with perceived addictive traits. The review highlights the critical importance of supporting children’s agency and digital citizenship skills from a young age, including the ability to critique content, balance screen-time with non-screen time and to develop self-control and self-regulation as a means to promote long-term positive outcomes for children in their digital lifeworlds and beyond.

## 1. Introduction

Young children’s increasing engagement with technology is an ongoing source of interest and often concern as technology’s ubiquity overlaps with pervasiveness. Discourse on the benefits and perils of digital technology in children’s lives has been at the forefront of discussion, both anecdotally and in research for well over a decade. Concerns about children’s use of digital technology have increased further due to the impact of COVID-19 and associated changes to the way society normally operates. Touchscreen technologies have featured strongly as a way to keep children engaged, and also entertained. It is expected that some of these changes will continue into a new normal as the world adjusts to post-pandemic life.

Even prior to the impact of COVID-19, screen time use by young children had grown due to the increased accessibility of Internet-enabled touchscreen devices. Lowrie and Larkin [1] note that the ease with which young children can use touchscreen devices has resulted in more engagement and also a wider range of digital play. Commonly, children begin engaging with apps and touchscreen devices as infants [2] and the Australian e-Safety Commissioner [3] recently reported that 94% of Australian children are accessing the Internet by four years of age. Other research indicates that the vast majority of children aged birth to 16 years now have access to a tablet (91%) or smartphone (86%) and engagement with digital content through viewing platforms such as YouTube is also common [4,5].

There are significant concerns about the content that young children are able to access through online viewing platforms as well as gaming apps. Nansen, Chakraborty, MacDougall and Vetere [6] note that these are largely unregulated spaces. There is an increasing presence of advertising, with many YouTube clips and games replete with branded products and content from commercial enterprises. The potential impact of advertising on children’s development is recognized by the Australian Communication and Media Authority and regulated through the Broadcasting Services Act 1992 and the 2009 Children’s Television Standards. Importantly however, the current guidelines and regulations do not apply to advertisements that appear online (as opposed to those on broadcast television). As an example, there is no requirement for advertising content to be clearly separate from gaming or online viewing content [6].

While reporting on digital wellbeing for adults, Ceccinato and colleagues [7] identify the power of digital content to attract and hold attention. This concern extends to children’s use of screen-based media with reports of children choosing to engage with screens over other activities or not being able to disengage from their touchscreen devices [8]. This is a highly contentious topic, often framed in terms of “technology addiction” and a lack of self-regulation. However, it is also an under-researched topic. It is common for young children to be so immersed in their play that they are unable to disengage easily when they are enjoying what they are doing [9,10]. To understand children’s experiences with technology, consideration must be given to whether digital play is viewed differently in this regard to more traditional forms of play.

This scoping paper examines the intersections between what is known about children’s immersion in play as well as digital play and the implications of this for children’s wellbeing. Flow theory provides a framework for defining this nexus, particularly when seeking to understand connections with habit formation and what can be seen as addictive behaviors. The paper also investigates gaps in knowledge identified in current research, such as the paucity of studies that look at the connection between children’s immersion in digital play and flow [10] and research investigating the association between engagement with digital technology and children’s wellbeing [11].

## 2. Materials and Methods

This paper is informed by a scoping review of current literature and information relating to young children and digital wellbeing. The study focuses on children in the prior to school years, as it is widely recognized that the children’s development in the years between birth and age five significantly exceeds their development at any other stage of the lifespan and remains an area that requires consistent reconceptualizing as the ubiquity of technology shapes a shifting society. Additionally, the early years are a time in which executive function and self-regulation skills develop rapidly, underpinning later capabilities [12]. A scoping review approach enables clarification and the definition of key concepts in emerging areas, particularly where there are inconsistencies or gaps in knowledge [13], as is the case with young children’s digital wellbeing. The methods section follows the five stages of a scoping review as recommended by Arksey and O’Malley [13].

### 2.1. Stage 1—Research Questions

The literature search aimed to address the overarching question of: What is known about digital wellbeing for children aged birth to five years? This question centered on the premise that children learn about and within their worlds through play [14] and that digital play is now an embedded and central part of young children’s everyday lives [15]. The specific research questions to be addressed in this paper include:

- What is known about children’s deep immersion in digital play and is it cause for concern?
- What is known about digital wellbeing for young children?
- What is needed to support young children’s digital citizenship and digital wellbeing?

### 2.2. Stage 2—Identifying Relevant Studies

Search criteria were developed to ensure that publications for review met the specific focus of the scoping paper. Initial searches of academic databases utilized ProQuest and EBSCO search platforms and included the terms “digital wellbeing” OR “digital well-being” AND “child*” (64 results). All but one of these related to primary school aged children, adolescents or older youths. To refine results to the age group relevant to this study the terms were adjusted to “digital wellbeing” OR “digital well-being” AND “early child*” (0 results) and “digital wellbeing” OR “digital well-being” AND “young child*” (44 results). Of these results only two sources were specifically related to children under the age of five, reinforcing the gap in available research.

Policy papers and other publications by recognized authorities were also sought to provide evidence of current thinking and recommendations. While these sources often do not appear in academic databases, they include research undertaken by large, esteemed bodies such as UNICEF and the OECD and are of contemporary salience. Search terms included: early childhood digital; technology addiction; technology self-regulation; flow and play; flow and technology; digital play.

### 2.3. Stage 3—Inclusions and Exclusions for Study Selection

The following criteria were applied to the database searches:

- Potential sources had to be peer-reviewed journal articles, book chapters or conference proceedings. Inclusion of peer-reviewed sources helped to ensure academic integrity [13].
- Publications by peak bodies were included, but these had to be recognized authorities with a robust research reputation (such as the OECD, UNICEF or national bodies such as Early Childhood Australia or the National Association for the Education of Young Children [NAEYC]).
- Results were limited to publications after 2011, and publications before this time were excluded. This was in recognition of the fact that iPads were released in 2009 and the increasingly widespread availability of touchscreen technology after this time led to a significant change in the way that young children engaged with the digital world [1].
- Initial searches aimed to identify studies that focused on digital wellbeing and children under the age of five (referred to in this paper as young children). This resulted in very few matches. The review was extended to include children in the six to 12 years age range (those attending primary/elementary school) with the aim of finding points of relevance to younger children and adapting.

### 2.4. Stage 4—Charting the Data

The synthesis process began with reviewing research articles and charting intersecting themes that emerged in a process of “sifting, charting and sorting” [12]. Articles were reviewed for key themes and content that specifically related to children’s immersion and engagement with digital technology, as well as elements that aligned with definitions of flow, play-based learning and elements of wellbeing. Article content was divided and reported across key themes and intersections that emerged.

### 2.5. Stage 5—Collating, Summarizing and Reporting the Results

Findings from the literature review are summarized to provide a synthesis of current information on young children’s experience with, and immersion in, digital play and how this informs the definitions and conceptualization of digital wellbeing. Flow theory is presented as an underpinning conceptual framework that provides academic rigor to the analysis.

### 2.6. The Conceptual Framework

The connections between Csikszentmihalyi’s flow theory [16] and play are evident, particularly in the early childhood years. Flow is defined by Csikszentmihalyi [16] as “the state in which people are so involved in an activity that nothing else seems to matter; the experience itself is so enjoyable that people will do it even at great cost, for the sheer sake of doing it” (p. 4). Parallels are evident between the characteristics of flow, and the characteristics of play-based learning: an approach that is at the forefront of contemporary early childhood pedagogy [14].

The concept of wellbeing is increasingly seen as a key measure of healthy development. Consideration of what wellbeing looks like in the context of young children’s digital play is limited, both in relation to the specific analysis of digital play itself, as well as more broadly in terms of the impact of digital play on a child’s overall sense of wellbeing. Given contemporary concerns about the “addictive” nature of technology and the impact of increased screen time on children’s development, a concept of “digital wellbeing” offers a way to consider how screen time and digital technology can be integrated into children’s lives in a way that has a positive impact on learning, development and long-term outcomes. Flow theory enables engagement in digital play to be considered alongside other forms of play to help build a greater understanding, particularly in relation to the deep immersion with digital technologies that can be seen as troublesome.

## 3. Results and Discussion

The literature search found a paucity of information relating to digital wellbeing for young children. While the literature indicated a connection between play and flow, and play and digital technology, these were not strongly represented in the research relating to children five years of age or under, despite their significant interactions with digital technologies. Digital wellbeing research was also not discussed in research findings relating to young children, but rather identified for older children and adults (for example [7,17,18]).

**Figure 1:**
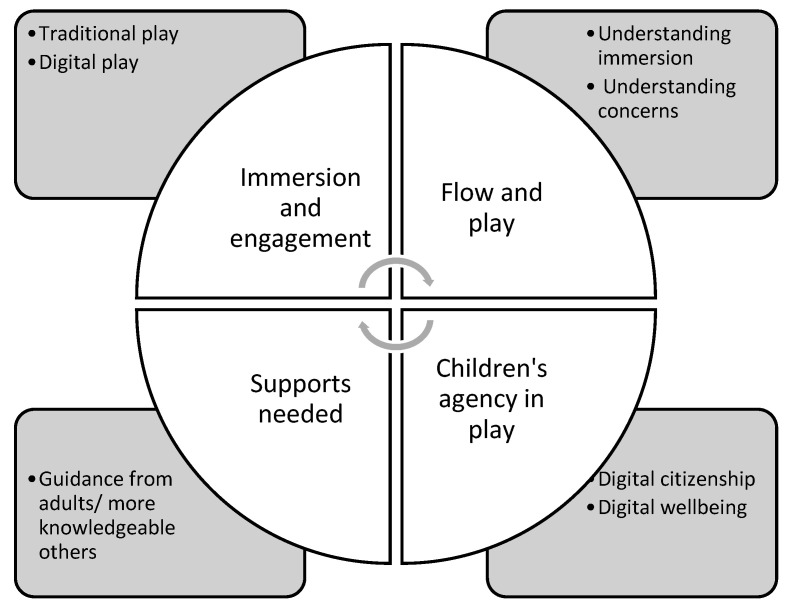
Matrix of key themes relating to digital play and digital wellbeing for young children.

Analyses of papers in the scoping review resulted in the identification of four key themes relating to digital play and wellbeing for young children, including: (1) immersion and engagement with technology; (2) flow and play; (3) children’s agency in play; (4) supports needed to help children develop digital citizenship skills and digital wellbeing such as self-regulation, safe technology use and balanced engagement with devices (see Figure 1). This section presents the synthesized results and discussion expanding on these four themes and drawing on flow theory as a conceptual framework.

### 3.1. Immersion and Engagement: Play/Digital Play

Play provides children with opportunities to make sense of their worlds and it also fosters knowledge of social and cultural practices and values [14,19]. While there is generally a strong understanding of play-based learning by those working with young children, this understanding is not necessarily shared by parents, family members, the broader community and the media, all of whom have a significant role in creating, shaping and perpetuating social norms. Elements of play, summarized from Gray [14] and Huizinga [20], include:

- Active—involves interaction with other people, resources or the surrounding environment
- Enjoyable—though can sometimes create frustration and challenges
- Sense of awareness that it is different from everyday life
- Symbolic in nature and sometimes only understood by the person playing
- Child led
- Voluntary
- Rules are accepted freely and respected as binding
- Able to control and direct actions
- Process based, focusing entirely on the activity rather than on an end product or achievement
- Intrinsic motivation
- Immersed in activity and less conscious of self and time

There are many diverse conceptualizations and definitions of technology and understandings of how and why it is relevant in young children’s play. While research has long reported on the presence of technology in play [21] the types of technology and the way that it features continues to evolve. Technology can afford new types of play [22], but it is often also assimilated into, and alongside, more traditional play [23]. For children, digital play is not necessarily identified as different from non-digital play and they often move between the two seamlessly [15].

A difference in the perceptions of technology and play/play-based learning between children and adults emerged as a theme from the literature search. Sulaymani and colleagues [22] report that school aged children in their study saw time with the iPads as play, where as teachers saw it as learning. This not only demonstrates the different motivations children and adults have around touchscreen devices, but also highlights that children do not make the distinction between play and learning in the same way that adults do. Furthermore, the differentiation between digital life and real life is becoming less distinct [24] and subsequently for many younger children “digital play” is just “play”.

As noted above, one of the key characteristics of play is its immersive quality. Regardless of the nature of the play they are engaged in children often become completely engrossed and find it difficult to leave their play or transition to other experiences [9]. Interruption to play is usually because of a competing demand and is often instigated by an adult [10]. This leads to an often-unwelcome disruption to the deep state of engagement that existed while the child was playing. While resistance to disengaging from play is not limited to screen-based play, difficulties in leaving digital play are often viewed as far more troublesome than those encountered in non-digital play. For example, a child who does not want to stop playing in a sandpit or with their Lego blocks is often viewed differently to one who does not want to leave their iPad or other device. The question arises as to why immersion in screen-based play is of greater concern. It is important to recognize that the aversion to young children’s screen use can often arise from a “moral panic” perspective. The technologies experienced by young children in contemporary society create vastly different childhoods to those experienced by their parents and families. This unfamiliarity with the relative newness of technology, particularly as it continues to shift and evolve, often impels wider concerns about the potentially insidious nature of technology use. As an example, a recent survey undertaken by the Royal Children’s Hospital in Melbourne, Australia [25] found that excessive screen time was rated by parents as the top health concern for children above other significant social issues such as bullying, unhealthy diets or mental health. It is also important to temper fear-based responses to technology use and to understand the conceptualizations and perceptions that underpin such perspectives. A key point of consideration is that while the value of play for children’s learning, health and wellbeing is becoming more widely documented, the value of free play is often underappreciated or not recognized [26]. This lack of appreciation is then magnified in the case of digital play which, due to its outwardly passive and seemingly solitary nature, is often seen as even less useful or valuable than traditional forms of play as a means for learning and development.

Another concern with digital technology relates to the virtual, and therefore potentially non-concrete, nature of the resources. Seminal research by Clements [27] identifies that as computers became more prevalent, debates emerged on whether manipulating digital resources provided the same sensory-concrete experiences for children as manipulating physical objects. Studies on block play with primary school aged children show that both physical and mental handling of blocks had a positive impact on learning, particularly in relation to mathematical skills [28,29]. Similarly, Sarama and Clements [29] report that the use of digital manipulatives had the potential to support children to build concrete knowledge by making their understandings explicit. Further to this, they identified that whether resources are physical or digital, the guidance and support received from educators is more important than the actual tools in developing children’s concrete thinking.

An additional concern identified in the literature review was that perceived passive engagement with touchscreen devices is often seen to replace active or physical play for young children. A wide range of recognized authorities report on the negative psychological and physiological impacts of passive screen use by children (such as the American Academy of Pediatrics [30], Early Childhood Australia [31], The Gonski Institute [32], OECD [18]) and correspondingly, research reinforces that physical activity benefits young children’s development in terms of cognitive and brain development, executive function as well as reducing anxiety and depression [32]. Connections are also evident between physical activity, executive function, attention and self-regulation [33] which have implications for children’s increasing use of touchscreen devices. However, active and passive engagement with technology is not a clear dichotomy. Games and even television style programs can be interactive in nature and form the impetus for social interaction (NAEYC and Fred Rogers Center for Early Learning and Children’s Media [34], OECD [18]). As an example, the app Pokémon Go requires children (and adults) to move around using GPS functions in touchscreen devices, or other apps that guide movement such as Just Dance Now or Toca Dance. As such, children’s engagement with ever-shifting digital technologies and screen media needs to be consistently critiqued and reconceptualized. It is acknowledged that children learn through play and that digital play is now a normal and embedded part of everyday life for young children.

### 3.2. Flow and Play: Understanding Immersion

A review of the literature also identified concerns around children’s intense immersion and engagement with touchscreen devices (such as [17]) and raised the question of why this was viewed differently by parents than immersion or engagement in other forms of play. Csikszentmihalyi [16] describes deep immersion as a state of flow. He explains that in this state “concentration is so intense that there is no attention left over to think about anything irrelevant or to worry about problems. Self-consciousness disappears, and the sense of time becomes distorted. An activity that produces such experience is so gratifying that people are willing to do it for its own sake, with little concern for what they will get out of it, even when it is difficult, or dangerous” (p. 71). When compared with the characteristics of play, a strong synergy can be seen with the eight elements of flow, as flow involves experiences that are borne from intrinsic motivation, that are enjoyable and led by the individual. Table 1 illustrates these connections and similarities.

### 3.3. Flow and Play: Understanding Concerns

The synthesis of play and flow state outlined in Table 1 demonstrates a clear connection between flow and play and provides insights into children’s experiences when they are fully engaged and present in their play. More specifically, Csikszentmihalyi [16] finds that many people playing games, and in particular board games, were in a state of flow. In later years, this approach has also been applied to digital games to better understand the deep engagement and investment people experience when playing (such as [35,36]).

Flow theory therefore provides a solid foundation from which to understand children’s enjoyment of digital games and how this relates to learning. From a theoretical perspective this raises positive and negative possibilities. A flow state involves a high degree of engagement and concentration and can foster positive developmental outcomes such as exploration and investigation skills. The balance between challenge and ability that is negotiated during a flow state is key to sustained engagement in play-based learning and the development of perseverance skills, both fundamental elements of effective learning [37,38]. Kiili and colleagues [38] extend Csikszentmihalyi’s [16] posits regarding the balance of challenge and achievement to include the zone of proximal development, that is, the child’s abilities and achievement when they have the support and guidance of a more knowledgeable other [39]. While flow state often presents as a solitary experience, the need for support and guidance, particularly in relation to digital technologies and young children emerges as a key point for consideration.

However, concerns about flow state and digital technology are also prevalent. Amos [40] referring to a study with older digital game players, identifies flow as a state that can induce the formation of habits and therefore have the potential to trigger addiction. Mavoa, Carter and Gibbs [41] in their study of primary school aged children’s engagement with Minecraft report that the term addiction was commonly used by many parents to explain their child’s immersion in the game. However, a further analysis of parent responses identified that this term was used when the child wanted to remain playing longer that the parent wanted them to. This aligns with findings in a UNICEF report on children, digital technology and wellbeing [42] which identifies issues with the term addiction being misused. The report notes that studies of children’s engagement with technology, and studies on technology and addictive behaviors are underpinned by vastly different research questions but that their findings are often inaccurately conflated by researchers and also by the media [42].

The term digital addiction remains subjective and ill-defined. There is no formal or clinical evidence of young children having technology addictions [42]. The inclusion of Internet Gaming Disorder in the Diagnostic and Statistical Manual of Mental Disorders (DSM) is controversial [43] and does not include a diagnosis of addiction. The models used to measure gaming disorders are those that are used for gambling machine addiction and relate to impulsive control disorders or behavioral addiction [42,43]. These include elements such as salience, euphoria, tolerance, withdrawal symptoms, conflict and relapse, and these are not recognized as characteristics present in digital gaming. However, elements such as euphoria, tolerance and cognitive salience can also be seen as indicators of intense engagement rather than just being related to addiction [43]. They can also be seen to align closely with the idea of “flow” as discussed above.

Not enough is known about these interactions and experiences to use the term addiction or to understand whether intense technology engagement is a precursor to addictive behaviors. Csikszentmihalyi [16] provides a perspective that assists in the understanding of children’s deep engagement with technology particularly in terms of enjoyment and a balance of challenge and ability. Perhaps this is the reason that young children find it hard to disconnect from play, whether that is the child who does not want to move away from painting at the easel or the child who is immersed in building a Minecraft world. It is also interesting to speculate on why deep engagement with some experiences might be viewed more positively than others. Again, conceptualizations of digital play, including negative perceptions of it as a solitary and passive experience need to be considered here.

### 3.4. Concerns—Corporatization and Connection with Flow

Increased technology has resulted in greater exposure to corporatized content. The research indicates that with increased access to screen-based technology children also have new identities as consumers and new ways of engaging in play [23]. Game and app developers are intentional in targeting children and in understanding how they can be positioned in the market. This can include the promotion of additional benefits as a subscriber (rather than just player/viewer) of a game or channel, or by gathering credits or rewards throughout gameplay that can be used to purchase items within the game [6]. Behavior theory shows a connection between rewards and habit formation [44] and this is a significant area for further exploration in defining and understanding digital wellbeing for children. It brings into question whether games or apps that have been commercialized in this way can still be identified as play-based learning. It may be that such games are no longer intrinsically motivating in the way that play is usually considered to be but rather extrinsically motivated by the rewards on offer.

Branding and advertising are prevalent in screen-based content and apps. Children can often recognize obvious advertising, but not necessarily embedded advertising [6]. As noted earlier, there are no laws on advertising content for Internet media. This is a significant issue, particularly given the increase in the accessibility of viewing platforms such as YouTube and YouTube Kids. However, advertising is rarely cited by families as a key area of concern in regard to online safety and wellbeing [6]. This disparity highlights the need to explore the impact of such advertising on young children further, both in terms of their perspective on it and the potential effects that such exposure might have on wellbeing.

### 3.5. Children’s Agency in Play: Understanding Digital Citizenship and Digital Wellbeing

The limited research on digital wellbeing focuses on primary aged children or older. Wellbeing itself can be difficult to measure and define in early childhood. Pollard and Lee [45] state there are no standard clinical measures for assessing the wellbeing of young children. However, the Leuven Involvement Scale for Young Children [46] provides a valuable guide for rating a child’s engagement that could underpin further consideration of digital wellbeing. The scale focuses on the role of the teacher and adult and the overall need for a child to feel a sense of belonging to achieve wellbeing. Laever [47] notes that when a child is at the “highest level of involvement; this is where deep-level-learning takes place” (p. 20), highlighting the potential for intense engagement in digital play to support learning and development in young children.

Aside from this, definitions of wellbeing for young children tend to focus on the negative aspects or what is missing, rather than the positive indicators of wellbeing [6,11]. This often includes a focus on the intersection of physical, mental, cognitive, social and economic domains [45]. Situating negative definitions and explanations of children’s wellbeing at the forefront is problematic because these position children as vulnerable rather than seeing their strengths and potential for agency [45]. Laever [47] presents wellbeing from a strength-based rather than a deficit perspective, providing action points that can help to improve children’s involvement, agency and sense of belonging, and therefore their wellbeing. On a broader scale, the OECD [48] has recently released a conceptual framework for measuring children’s wellbeing. Of key importance is that the new model looks at diverse factors that influence wellbeing such as children’s relationships, their environments and other experiences, and aims to include children’s voices in the process of measuring and understanding wellbeing [48] These wellbeing measures provide essential considerations for further developments of the concept of digital wellbeing for young children.

It can be extrapolated that understanding the factors that create deep immersion are key to supporting digital wellbeing. Reports indicate that young children are often not able to disengage easily or that it negatively affects their mood or behavior. However, the focus must extend beyond this to help children develop a sense of agency in their use of digital technology and to build the ability to self-regulate. Self-control relates to being able to show restraint regarding impulses, whereas self-regulation is more complex and involves being able to acknowledge the cause of impulses, take measures to minimize the intensity and when needed being able to actively resist [49]. For young children this begins with self-observation, followed by self-judgement and self-reaction [50]. Consideration of young children’s self-regulation in digital spaces must include a strong understanding of these developmental pathways, both in terms of the expectation placed on children’s behavior as well as in terms of considering the guidance and support required. Further research is needed to investigate and understand the impact of digital media on children’s nervous systems and how this then affects their ability to self-regulate. Young children are often not able to express states of hyperarousal, and instead it will show in their behavior. It is important for parents, families and educators to be able to recognize the signals and support effective management [49]. Further understandings in this area are an important underpinning factor of developing measures to support children’s digital citizenship and digital wellbeing.

The research reinforces that rather than supervising and protecting children in digital spaces there is a need to scaffold and support them in ways that will build independence, agency and empowerment [6]. Chayko [24] identifies a need to understand more about people’s capacity for agency in digital spaces. Young children are perhaps the most overlooked when considering this issue. Pivotal to this approach is having high expectations of young children’s capabilities but also not overstating or assuming knowledge. Children’s interest in or engagement with technology does not necessarily correspond with a deep or sophisticated understanding [51]. It is therefore important to ensure that educators and family members have an accurate understanding of children’s abilities as well as of the potential dangers and benefits of digital play [6,9]. Achieving the balance between high expectation and not overestimating ability means that the adult needs to make time to talk through the children’s engagement with digital technology. Engaging in discussion with children can enable adults to build an understanding of the child’s ability and to purposefully determine how to best guide and scaffold the development of elements that will support wellbeing such as balancing screen time with non-screen time, critiquing content, self-control and self-regulation. There is also a requirement for adults to understand the technology themselves so that they are able to effectively evaluate and guide [52,53].

Digital wellbeing therefore overlaps with digital citizenship, a term that is more familiar in relation to young children’s digital experiences. The NAEYC and Fred Roger’s Center [36] position statement on technology and interactive media for children birth to eight years of age defines digital citizenship as: “[…] The need for adults and children to be responsible digital citizens through an understanding of the use, abuse and misuse of technology as well as the norms of appropriate, responsible and ethical behaviors related to online rights, roles, identity, safety, security and communication” (p. 10). This definition reinforces the need to support more than just technology handling skills for young children. There also needs to be a focus on active use and critical engagement [6]. Guidance and support from adults can result in cognitive benefits and self-regulation in children’s digital play and engagement, but again this needs to be purposeful, thoughtful and informed. Central to this process is building children’s skills and agency. As an example, where there is concern about over engagement, a response that just focuses on removing or limiting screens does not enable children to develop the necessary strategies to self-manage time and engagement [7]. Similarly, while adult controlled filters or restrictions or even co-viewing with a child can support safety, they do not empower children to build self-regulation skills [6]. Instead, effective digital citizenship can be supported through building young children’s abilities to critique and deconstruct content and to self-regulate their engagement and behavior. Central to building these skills is a focus on play-based learning and on maintaining and bolstering children’s agency.

### 3.6. Moving Forward—Understanding Digital Wellbeing

It is of high importance that families, educators and the wider community work together to build an understanding of digital literacy and citizenship that highlights their importance for young children’s development in a digital world while also recognizing the potential impact such digital engagement may have on children’s wellbeing. Underpinning this process is the understanding that moral panic responses are not new. Mavoa and colleagues [41] draw on research from the 1960s, where people were exceedingly concerned about the influence of television on children. Here the “addiction” fear related to the fact that television viewing was time consuming and distracted children from reality. There was a fear that withdrawal was a precursor to addiction. To overcome such concerns and create more productive pathways for engaging with technology there need to be clear understandings of the synergy between factors that shape beliefs and responses to children’s engagement with technology [51].

It can be surmised from the literature review that rather than restricting or removing technology, it is important for adults to engage children in conversations about their online play. Lowrie and Larkin [1] note that this should be a continuous process with discussions taking place during screen time as well as before and after. Mavoa and colleagues [41] report that families rarely talked with their children about technology engagement but still held onto fears of “addiction”. Moving forward there is a need for research with children where they share beliefs and perspectives on their digital experiences and their impact on wellbeing [54], rather than just adult perspective which often predominate [55]. This could also include the observation of young children’s interactions with digital devices, such as the approach employed by Danby and colleagues [2]. Further research should focus on embedding and understanding children’s perspectives along with understandings of how parents and other adults can be supported to foster children’s digital citizenship and promote digital wellbeing. Guidance is pivotal in promoting the safe and appropriate use of technology while also creating awareness of associated risks [17].

Additionally, there is a need to work with the designers responsible for developing apps and games for young children. Tenets of flow theory have value for the development of high-quality digital content that supports learning and development [40,41]. Beyond positive learning outcomes, collaboration with developers and designers of apps and games for children must ensure their accountability in terms of children’s rights as digital citizens, including children not being misled and exploited and their right to safety in digital spaces being upheld.

## 4. Conclusions

This scoping paper provides insights into factors that influence conceptualizations of digital technology use by young children. This paper proposes that digital play is not understood, and that it is met with greater aversion that other forms of play. Flow theory [16] is presented as an effective theoretical framework through which to interrogate young children’s engagement and immersion with digital play and devices. While children’s deep immersion in digital play often sparks concerns in families and in broader communities [56], understanding the elements that influence this immersion is key to supporting digital wellbeing. A connection between digital wellbeing and citizenship is also identified. Further research should examine the ways to support children to become agentic in digital spaces, including the ability to deconstruct and critique information and to self-regulate behaviors. Effective guidance and support from parents, families and more knowledgeable others is pivotal in this process. Developing definitions and conceptualizations of digital wellbeing can focus on positive aspects and the value it can afford children rather than what it looks like when that wellbeing is absent. It is proposed that further research that examines children’s deep engagement through the lens of flow theory would help to build deeper understandings of digital play. This paper also identified the value of embracing synergies between theoretical perspectives to build holistic understandings. Employing a range of theoretical lenses can enable deep and critical understandings of children’s experiences. It is of critical importance that young children are supported to develop critical thinking skills, self-regulation and agency with digital technology from an early age. Adults must be able to guide and support young children as they develop these skills, which will require the development of support and interventions not only for young children, but also for the adults in their lives. A limitation of this scoping review is that articles relating to young children’s experiences in digital spaces are rarely from their perspectives and instead rely on interpretation from adults. Further research that includes children’s perspectives on digital participation is needed to develop authentic strategies for supporting children’s digital wellbeing.

## Figures and Tables

**Table 1:** Shared characteristics of a state of flow and play.

Flow—Csikszentmihalyi [16]	Play—Gray [14], Huizinga [20]
Clear sense of what needs to be done moment by moment	Certain rules or limitations associated with when and where it takes place
2. Immediate feedback on how well one is doing	Active—involves interaction with other people, resources or the surrounding environment
3. Intense concentration of attention	Process based, focusing entirely on the activity rather than on an end product or achievement
4. Balance between action (challenge) and capacity to act (skills)	Enjoyable—though can sometimes create frustration and challenges
5. Able to ignore irrelevant external content—remove from consciousness	Sense of awareness that it is different from everyday life Symbolic in nature and sometimes only understood by the person playing
6. Sense of control over the activity	Play is voluntary Child-led Rules are accepted freely and respected as binding Able to control and direct actions
7. Distortion of sense of time	Less conscious of self and time
8. Intrinsically rewarding- doing it for its own sake	Process based Intrinsic motivation

## Data Availability

No new data were created or analyzed in this study. Data sharing is not applicable to this article.
